# Significance of the organic aerosol driven climate feedback in the boreal area

**DOI:** 10.1038/s41467-021-25850-7

**Published:** 2021-09-24

**Authors:** Taina Yli-Juuti, Tero Mielonen, Liine Heikkinen, Antti Arola, Mikael Ehn, Sini Isokääntä, Helmi-Marja Keskinen, Markku Kulmala, Anton Laakso, Antti Lipponen, Krista Luoma, Santtu Mikkonen, Tuomo Nieminen, Pauli Paasonen, Tuukka Petäjä, Sami Romakkaniemi, Juha Tonttila, Harri Kokkola, Annele Virtanen

**Affiliations:** 1 Department of Applied Physics, University of Eastern Finland, Kuopio, Finland; 2 Atmospheric Research Centre of Eastern Finland, Finnish Meteorological Institute, Kuopio, Finland; 3 Institute for Atmospheric and Earth System Research/Physics, Faculty of Science, University of Helsinki, Helsinki, Finland; 4 Aerosol Physics Laboratory, Physics Unit, Tampere University, Tampere, Finland; 5 Department of Environmental and Biological Sciences, University of Eastern Finland, Kuopio, Finland; 6 Institute for Atmospheric and Earth System Research/Forest Sciences, Faculty of Agriculture and Forestry, University of Helsinki, Helsinki, Finland

**Keywords:** Atmospheric science, Atmospheric chemistry, Climate change

## Abstract

Aerosol particles cool the climate by scattering solar radiation and by acting as cloud condensation nuclei. Higher temperatures resulting from increased greenhouse gas levels have been suggested to lead to increased biogenic secondary organic aerosol and cloud condensation nuclei concentrations creating a negative climate feedback mechanism. Here, we present direct observations on this feedback mechanism utilizing collocated long term aerosol chemical composition measurements and remote sensing observations on aerosol and cloud properties. Summer time organic aerosol loadings showed a clear increase with temperature, with simultaneous increase in cloud condensation nuclei concentration in a boreal forest environment. Remote sensing observations revealed a change in cloud properties with an increase in cloud reflectivity in concert with increasing organic aerosol loadings in the area. The results provide direct observational evidence on the significance of this negative climate feedback mechanism.

## Abstract (web summary)

Vegetation emits organic vapors which can form aerosols in the atmosphere and influence cloud properties. Here, the authors show observational evidence that warmer temperatures lead to increased emissions of these aerosols in boreal forests which cause surface cooling, demonstrating a negative climate feedback mechanism.

## Introduction

On global scale, organic aerosol (OA) forms one of the major constituents of atmospheric aerosol particles^1^. Aerosol particles affect Earth’s radiation budget directly by scattering solar radiation, and indirectly by acting as a cloud condensation nuclei^2,3^, therefore the significant role of organic aerosols in the climate system is evident. As climate changes, climate feedback mechanisms either strengthen or lessen the change. Particularly, in atmosphere–biosphere feedback mechanisms, the biosphere responds to changes in atmosphere and this response in turn influences the climate. One specific driver for atmosphere–biosphere feedbacks is the strong positive response of the emissions of biogenic volatile organic compounds (BVOC) from vegetation to increasing temperature^4–6^. Then, the enhanced formation of secondary organic aerosol from BVOCs through chemical reactions is suggested to increase not only the aerosol mass but also the number concentration of particles large enough to act as cloud condensation nuclei (CCN)^7^. The increase in CCN number concentration is due to the enhanced condensational growth of particles which increases their probability of reaching sizes large enough to act as CCN instead of being scavenged by coagulation^8,9^, and potentially also due to enhanced nucleation^10^. Increased CCN number concentration, on the other hand, has been shown to produce smaller cloud droplets and brighter clouds^11–13^, leading to less solar radiation reaching the Earth’s surface. Hence, biogenic secondary organic aerosol (BSOA) is expected to have negative climate forcing both through the direct and indirect aerosol climate effects. This “BSOA-driven feedback” has been suggested to form a negative climate feedback particularly in the wide boreal forest regions where BSOA is an important constituent of ambient aerosol and influence of anthropogenic pollution is low^14,15^. There, the emissions of the main BSOA precursors, monoterpenes, and sesquiterpenes, increase exponentially with temperature^4,16^ and, consequently, enhanced BSOA formation may be expected in a warming climate.

Previous investigations have shown evidence of different steps of the BSOA-driven feedback. Paasonen et al.^17^ observed increasing number concentration of CCN-sized particles with increasing temperature at several measurement sites, while direct evidence on the link to organic aerosol formation lacked, and some previous studies report also an increase in aerosol optical thickness with temperature above forested area^18,19^. When these steps are put together in climate models, simulations predict such feedback^14^.

Here, we show that BSOA may form a significant atmosphere–biosphere feedback mechanism in a boreal forest environment. Our results provide observation-based evidence for full BSOA-driven feedback cycle starting from temperature-driven increase in organic aerosol mass, continuing to increasing CCN concentration, and finally to cloud physical and radiative properties.

## Results

### Relation between temperature and OA

**Fig. 1:**
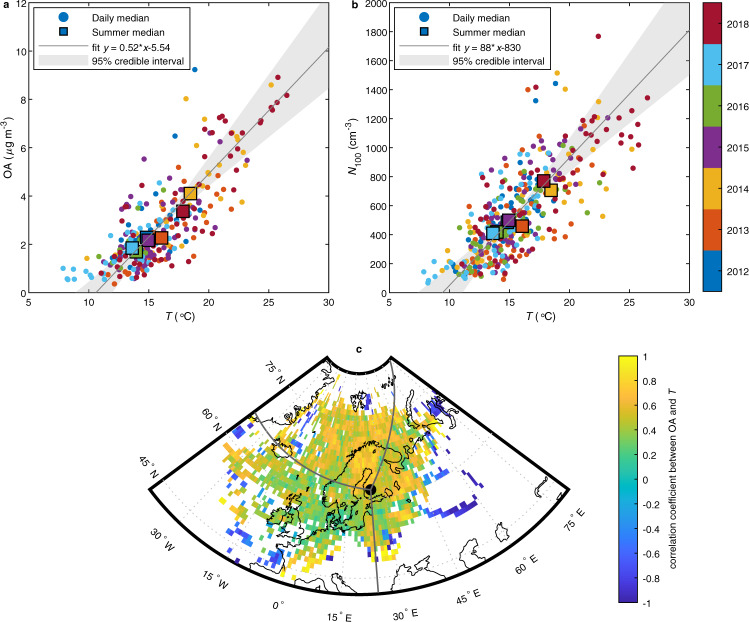
Field observations on changes in organic aerosol (OA) mass loading and number concentration of cloud condensation nuclei-sized particles with temperature (*T*). **a** OA mass concentration as a function of temperature. **b** Number concentration of particles larger than 100 nm (*N*_100_) as a function of temperature. Daily medians for summer (July–August) are shown with circles and summer medians with squares. The marker color indicates the year. Fitted Bayesian linear regression model is shown with gray line with the shaded area indicating the 95% credible interval. **c** Correlation coefficient between OA and temperature separated by the origin of air masses. The lines indicate the limits of the clean, eastern, and southern sectors.

We investigate the “BSOA driven feedback” at a boreal site by studying the relationship between organic aerosol loadings of biogenic origin and temperature, and further the consequences on direct and indirect aerosol effects using field and remote sensing observations. In our analysis, we utilized long-term aerosol composition data measured with an Aerosol Chemical Speciation Monitor^15,20^ (ACSM) at Hyytiälä Forestry Field Station, SMEAR II, in Finland^21^. During the analyzed 7-year period, from 2012 to 2018, average July–August temperature varied by ~5 °C and the period included two summers that were considerably warmer than the 20 years is 1999–2018. We have limited the analysis to two summer months (July–August) each year to minimize the effects from seasonal variation in vegetation, and to highlight the temperature-driven changes in organic loadings. As shown in Fig. 1a, there is a clear increase in organic mass loadings with increasing temperature. This trend is visible consistently with summer, daily, and hourly averages. OA concentration increased by a factor of 2.2 with the 4.9 °C increase of summer average temperature. Organics account for majority of the aerosol mass during summer at this location^15^ and, thus, the total particle mass concentration increases with temperature as well (Supplementary Fig. 1).

Similarly to the OA mass concentration, the number concentration of particles large enough to act as CCN, here defined as particles with diameter larger than 100 nm (*N*_100_), increased with temperature, consistently with previous observations^17^ (Fig. 1b). This is in line with intensified production of CCN-sized particles from secondary aerosol formation through enhanced particle growth and/or nucleation due to organics^7,22^.

Using Bayesian linear regression on the summertime daily medians we found an increase of 0.52 μg m^−3^ °C^−1^ (95% credible interval 0.40–0.69 μg m^−3^ °C^−1^) in the OA mass loading with temperature (Fig. 1a). Corresponding increase in *N*_100_ was 88 particles cm^−3^ °C^−1^ (95% credible interval 66–119 particles cm^−3^ °C^−1^) (Fig. 1b). It should be noted that the column burdens of OA and *N*_100_ may have a stronger increase with temperature compared to the change in observed surface level concentrations since the planetary boundary layer on average extends higher on warmer days^23^. The increase of both OA and *N*_100_ with increasing temperature indicates negative direct and indirect radiative forcing feedbacks to increasing temperature over summertime.

Warm and cold summer temperatures may be associated with different synoptic situations and, consequently, with different source areas for air masses arriving to the site. Therefore, the contribution of temperature-enhanced BSOA production on changes in OA mass loading needs to be distinguished from the effects of variations in air mass source area and contributions of the anthropogenic or biomass burning emissions^14^. To investigate if the increase in OA mass loading is dominated by increased BSOA production we utilized 96-h air mass back-trajectories calculated with HYSPLIT^24^ as well as in situ observations of black carbon (BC) and trace gases. Northwesterly-northern direction (280–30°) is considered as a clean sector with little anthropogenic influence for air masses arriving to this site^25^. Air masses arriving from eastern (30–180°) and southern (180–280°) sectors are subject to more anthropogenic emissions and especially the former also to biomass burning emissions^18,26^. The air masses from the eastern sector were on average associated with both higher OA mass loadings (median 4.8 μg m^−3^) and higher temperatures (median 18.7 °C) compared to air masses from the clean (1.1 μg m^−3^, 12.6 °C) and southern (2.3 μg m^−3^, 15.5 °C) sectors (Supplementary Figs. 2 and 3c, d). The southeastern arrival direction was also emphasized in air mass source areas on the warmer summers 2014 and 2018 compared to the rest of the summers (Supplementary Fig. 3). However, our analysis shows that OA mass loading and temperature observed at Hyytiälä correlate positively regardless of the source area of the air mass (Fig. 1c). This indicates that the temperature dependence in OA mass loading is primarily driven by enhanced BSOA formation with increasing temperature and not by the origin of the air masses. This conclusion is also supported by the statistical analysis using multivariate mixed-effect model^27^ (Supplementary Table 1, see Supplementary Discussion).

Aerosol concentration at Hyytiälä has been shown to increase as a function of the time over land, i.e., time subject to potential BVOC emissions, prior to arriving at the site from the clean northwestern sector due to the larger accumulated BVOC emissions^28,29^. This effect is visible, although not dominating, also in our OA mass loading observations (Supplementary Fig. 4). Our data show that for equal time over land, OA mass loading increases with temperature (Supplementary Fig. 4a, c). The data presents also signs of particle wet removal as higher OA mass loadings were observed when air mass had been subject to little or no rain and correspondingly OA mass loadings were lower when air mass had been subject to highest amounts of rain (Supplementary Fig. 4b, d). Nevertheless, the increasing trend of OA with temperature exists also when analyzing only air masses that have experienced no rain over the last 96 h. Neither time over land nor the cumulated rain along the back-trajectory correlates with temperature, which further supports the conclusion that the observed increase of OA mass loading with temperature cannot be solely explained with these air mass history-related factors.

To conclude, our analysis shows that the observed trend is affected by several factors, but it is dominated by the temperature increase. Based on the analysis done by using multivariate mixed-effect model the estimated temperature-dependent increase in OA mass loading was 0.24 μg m^−3^ °C^−1^ (95% confidence interval 0.22–0.25 μg m^−3^ °C^−1^). This suggests that the temperature-driven effect on OA is approximately half of the value interpreted directly from the slope between OA mass loading and temperature.

### Aerosol direct radiative feedback

**Fig. 2:**
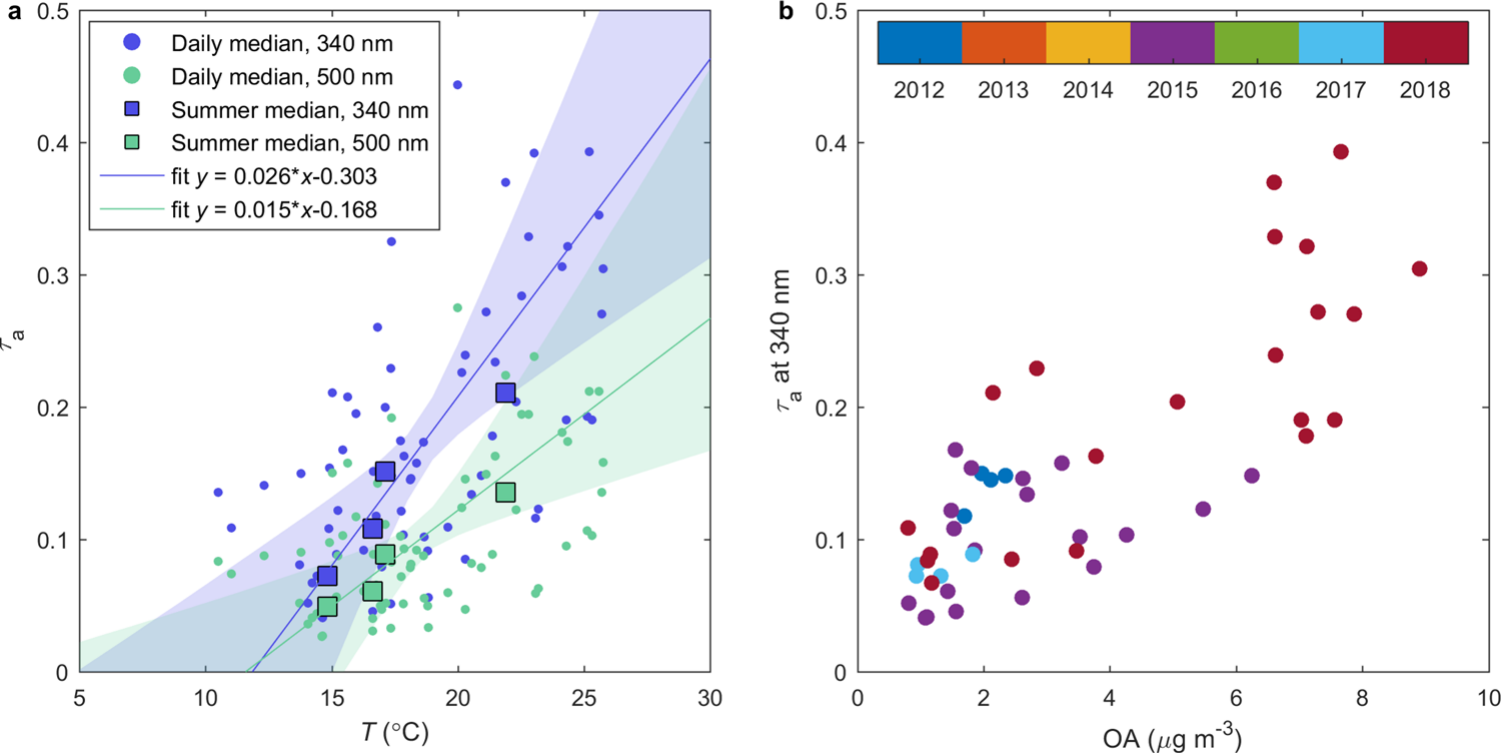
Aerosol optical thickness (*τ*_a_) measured with sun photometer at Hyytiälä. **a***τ*_a_ at 340 nm (purple) and 500 nm (green) as a function of temperature (*T*). Both daily (circle) and summer (square) medians are shown. Summer median *τ*_a_ are included only for years 2012, 2015, 2017, and 2018 due to data availability. **b** *τ*_a_ at 340 nm as a function of organic aerosol (OA) mass loading. Daily medians are shown with color indicating the year.

To investigate the effect of increased OA loadings on the aerosol direct radiative feedback (DRF), we analyzed aerosol optical thickness (*τ*_a_) observations available from an Aeronet Sun photometer and MODIS-Aqua satellite-instrument^30,31^. Aerosol optical thickness is a measure of how much aerosols prevent light from traveling through the atmosphere. The sun photometer observations revealed an increase in clear sky *τ*_a_ with increasing temperature over the measurement station. Figure 2a shows the increasing trend with temperature in *τ*_a_ measured at both 340 nm (slope 0.026 °C^−1^ (95% credible interval 0.013–0.047 °C^−1^)) and 500 nm (slope 0.015 °C^−1^ (95% credible interval 0.006–0.031 °C^−1^)) wavelengths. Moreover, the increase in both wavelength dependence of *τ*_a_ as described by the Ångström exponent parameter and the fine mode *τ*_a_ with increasing temperature suggest that the contribution of smaller particles on *τ*_a_ increases with temperature (Supplementary Fig. 5). This indicates that a significant contribution to the temperature trend in *τ*_a_ is coming from regional sources, e.g., BSOA, instead of long range transported (larger) aerosol particles. Furthermore, similar increase of *τ*_a_ with temperature is found with satellite observations (Supplementary Fig. 5). The positive correlation between *τ*_a_ at 340 nm over the measurement site and OA mass loading at the site (Fig. 2b) further supports the hypothesis that BSOA is the main cause for the observed temperature trend of *τ*_a_.

Based on the observed linear relationships between *τ*_a_ and temperature we estimated the regional DRF of the temperature-dependent *τ*_a_ component for clear- and all-sky conditions^32^. The sun photometer-based (at 500 nm) DRF estimates for Hyytiälä are −1.15 W m^−2^ °C^−1^ (95% credible interval −2.49 to −0.47 W m^−2^ °C^−1^) and −0.33 W m^−2^ °C^−1^ (95% credible interval −0.72 to −0.14 W m^−2^ °C^−1^) for clear- and all-sky conditions, respectively. The magnitudes of corresponding DRF estimates from MODIS retrievals are slightly larger (−1.56 W m^−2^ °C^−1^ clear sky, −0.45 W m^−2^ °C^−1^ all sky), however, they fall within the uncertainty of sun photometer-based estimates (Supplementary Table 2). The all-sky DRF estimates are in the same range as estimated for mixed forests in Southeastern USA (−0.5 ± 0.3 W m^−2^ °C^−1^)^33^.

### Cloud albedo feedback

**Fig. 3:**
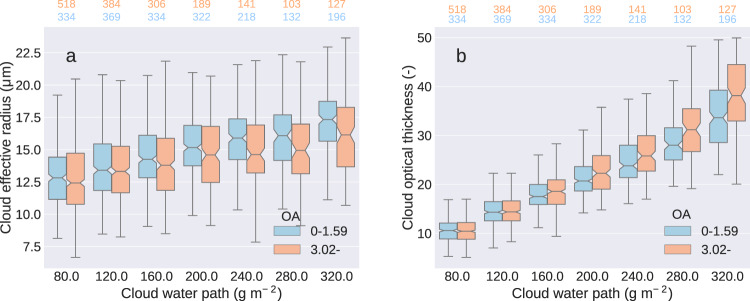
Cloud properties and organic aerosol mass loading. **a** Cloud effective radius and **b** cloud optical thickness divided based on the level of cloud water path. Data are divided to low (<33rd percentile (1.59 μg m^−3^), blue) and high (>66th percentile (3.02 μg m^−3^), red) organic aerosol (OA) mass loadings. The box shows the quartiles of the dataset while the whiskers show the rest of the distribution, except for points that are determined to be “outliers” using a method that is a function of the inter-quartile range. The notch in the box displays the confidence interval around the median. The blue and red numbers above each figure indicate the number of data points in each box.

We investigated the effect of enhanced BSOA formation to the cloud albedo effect of aerosols by analyzing the cloud effective radius (*r*_eff_) and cloud optical thickness (*τ*_c_) (the measures of cloud droplet size and the reflectance of clouds, respectively) retrieved from the MODIS-Aqua observations over southern Finland (land regions between latitudes 60° and 66° and longitudes 22° and 30°). As the temperature is strongly related to overall meteorological conditions, the connection between OA mass loading and cloud properties may be masked by variations in cloud type. Therefore, the analysis was limited to liquid clouds (cloud top temperature over −15 °C) and performed for seven categories of cloud water path, i.e., amount of water in cloud droplets in an atmospheric column. The data were divided into two groups based on the amount of OA. The OA mass loading was considered low if it was below 33rd percentile and high if it was above 66th percentile of the values. As seen in Fig. 3, both *r*_eff_ and *τ*_c_ increase with increasing cloud water path. However, at a same level of cloud water path, *r*_eff_ is smaller and *τ*_c_ higher at high OA mass loadings compared to low OA mass loadings. The differences between the loading classes are statistically significant (*t*-test with 95% confidence level) in the five highest cloud water path categories. Corresponding differences in *r*_eff_ and *τ*_c_ are observed if the data are divided into two classes based on *N*_100_ (Supplementary Fig. 6). This result is consistent with the hypothesis of enhanced BSOA formation increasing the number concentration of CCN which in turn leads to smaller cloud droplets and more reflective clouds. Consequently, these satellite observations show a strengthening of the indirect aerosol climate effect with increasing temperature and organic aerosol loadings in the area.

From the statistically significant changes in *τ*_c_ between high and low OA data, we estimated the corresponding change in cloud albedo and further the cloud albedo effect (CAE) which was −1.87 W m^−2^ (95% confidence interval −3.63 to −0.26 W m^−2^) (see “Methods”, Eqs. (2) and (3)). The albedo change corresponds to an average increase of 3.3 μg m^−3^ in OA mass loading, which in turn corresponds to a temperature difference of 6.3 °C since the OA mass loading was shown to increase by 0.52 μg m^−3^ °C^−1^. Consequently, the temperature-dependent cloud albedo feedback is −0.30 W m^−2^ °C^−1^ (95% confidence interval −0.58 to −0.04 W m^−2^ °C^−1^), approximately the same as the all-sky direct aerosol feedback. With the temperature dependence of OA mass loading indicated by the multivariate mixed-effect model, i.e., 0.24 μg m^−3^ °C^−1^, the OA mass loading increase of 3.3 μg m^−3^ would correspond to temperature difference of 13.8 °C, indicating a cloud albedo feedback of −0.14 W m^−2^ °C^−1^ (95% confidence interval −0.27 to −0.03 W m^−2^ °C^−1^).

## Discussion

**Fig. 4:**
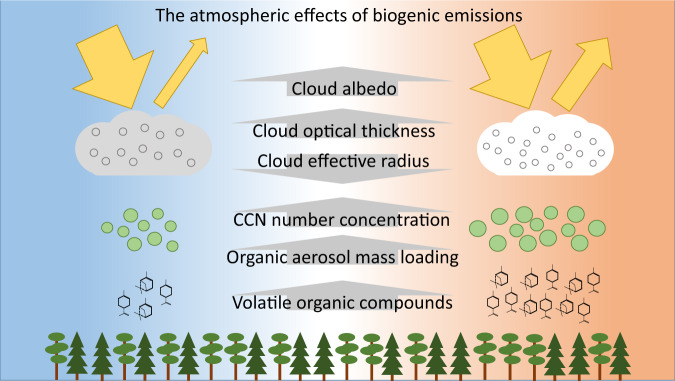
Temperature-dependent effect of biogenic secondary organic aerosol (BSOA) on cloud properties. In warmer conditions (on right), volatile organic compound (VOC) emissions from vegetation are higher compared to colder condition (on left). Consequently, more VOC are available for oxidation and forming BSOA. This leads to higher organic aerosol mass loading and higher cloud condensation nuclei (CCN) number concentration. These in turn lead to higher number but smaller size of the cloud droplets, increased cloud optical thickness, and consequently to stronger aerosol indirect effect on climate.

The results presented here provide direct evidence of the effect of temperature-dependent BSOA formation on the direct and indirect climate effects of aerosols, based on long-term OA measurements. Our estimates of direct and cloud albedo feedbacks are likely upper limit estimates as they are based on the total change with temperature and, as seen with OA mass concentration and estimated for cloud albedo feedback, the real temperature dependence may be somewhat weaker. Our analysis was based on observations from summertime when the precursor concentrations and likely also BSOA-driven climate feedback are at their annual maximum^16,17^. Consequently, the estimates of the strength of the feedback presented in this study are not equivalent to annual estimates derived, e.g., from models^14^. The spatial representativeness of the observations is challenging to estimate as it is affected by both the land use, i.e., dominant vegetation and their emissions, and the meteorology, i.e., cloud cover and dominant cloud types. In terms of vegetation, Hyytiälä is located on the area dominated by evergreen needleleaf trees which cover a major part of European boreal forest and wide areas in North American boreal forest^34^. Climate change also leads to changes in vegetation^35^ which may affect BVOC emissions and furthermore BSOA formation. As far as the current anomalously warm summers can be used as simulations of the future warming climate, our results indicate a significant negative feedback from BSOA via strengthening of both direct and cloud albedo effects of aerosols (Fig. 4). Taking the sum of the estimated cloud albedo feedback and the sun photometer-based (500 nm) estimate for the direct radiative feedback gives combined feedback of −0.63 W m^−2^ °C^−1^. Therefore, in areas represented by this boreal forest site the estimated combined enhancement in aerosol direct forcing and cloud albedo effect per 1 °C temperature increase is ~18% of the current effective radiative forcing of anthropogenic aerosols over boreal forest area in summer (see “Methods”). Therefore, if temperature increases by 3 °C (in accordance with the intermediate stabilization pathway RCP4.5 scenario^36^), this BSOA-driven feedback may strengthen the aerosol radiative forcing by approximately half of the present-day summertime aerosol radiative forcing in boreal area. There are currently large variability in simulated BSOA climate effects between Earth system models^37^ and our results demonstrate the significance of further developing the representation of BSOA-driven climate feedback in the models, including precursor emissions, yields and properties of condensable oxidized organics, aerosol population dynamics, and aerosol–cloud interactions.

## Methods

### Field measurements

Field measurements were carried out between 2012 and 2018 at SMEAR II station at Hyytiälä, Finland, which is a boreal forest background station with surroundings dominated by Scots pine forest^21^. Particle number concentration was measured with differential mobility particle sizer (DMPS)^38^ which samples at ca. 8 m above ground. Sub-micrometer non-refractory aerosol chemical composition was measured with an Aerosol Chemical Speciation Monitor (ACSM) which provides concentrations of particle-phase organics, sulfate, ammonium, nitrate, and chloride^15,20^. Inlet of ACSM is at a height of ca. 4 m above ground. Temperature was measured at 16.8 m above ground with Pt100 sensor. In addition to the number concentration of particles larger than 100 nm (*N*_100_), we analyzed the CCN number concentration data measured at 8 m above ground with CCN counter at 0.2% supersaturation from years 2013 to 2018^39,40^. CCN data had low time resolution of 4 h and the data were missing for large part of summers 2013 and 2016 and therefore main analysis was based on *N*_100_ from DMPS data instead. Supporting field measurements were black carbon (BC) concentration measured with an aethalometer at 880 nm wavelength for 2013–2018^41^, wind direction from the above canopy, global radiation intensities at 18 m above ground until 2016 and at 35 m from 2017, carbon monoxide (CO) concentration at 16.8 m and NO_x_ concentration averaged over measurements at seven heights between 8.4–125 m due to data availability.

### Analysis of the field data

Hourly means were calculated from all field data and this hourly data were used as the base dataset for the analyses. Hourly data points with wind direction between 120° and 140° were disregarded from the dataset to exclude the influence of the emissions from a nearby saw mill^42^. Daily medians were calculated based on the hourly data by averaging from midnight to midnight local winter time (UTC + 2). Each day was divided into six consecutive 4-h time windows starting from midnight and a daily median of a variable was accepted in the analysis only if at least one data point existed for the variable for each of the 4-h time windows. This was done to ensure that the daily median value would not present only some part of the day in case of discontinuous data. The studied time period extends over seven summers and was chosen based on the extend of the ACSM measurements. Coverage of ACSM data was good for summers 2015, 2017, and 2018, however, gaps with data missing for 5 or more days occurred during the remaining summers on the following periods: 25.7.–23.8.2012, 9.8.–31.8.2013, 16.8.–31.8.2014, and 1.7.–12.8.2016. Due to these discontinuities in the data, averaging over the whole of July and August each year would lead to inconsistencies in summer medians of the different variables. Therefore, the summer medians for OA, *N*_100,_ and temperature in Fig. 1 were calculated based on only those hourly data points for which data for all three variables were available. This way the summer medians of the three variables all represent a common time period for a particular year.

While the OA mass concentration exhibits increase with temperature, the increase of organic fraction with temperature is less apparent (Supplementary Fig. 1). Consistently, also concentration of particulate sulfate and nitrate shows an increase with temperature (Supplementary Fig. 1). However, organics constitute the majority of the measured particulate compounds during summer in Hyytiälä.

A brightness parameter (BP) value was calculated as a ratio between the measured global radiation and the theoretical maximum global radiation intensity and data were divided based on the calculated BP into “cloudy” (BP < 0.3) and “clear sky” (BP > 0.7) cases^43^ (Supplementary Fig. 8, see Supplementary Discussion).

### Analysis of air mass origin based on trajectories

HYSPLIT (Hybrid Single-Particle Lagrangian Integrated Trajectory)^24^ model was used for calculating 96-h back-trajectories for air masses arriving in Hyytiälä with 1 h resolution at the height of 100 m above ground. A precompiled Linux version of the HYSPLIT model (revision 854; obtained from the National Oceanic and Atmospheric Administration (NOAA) Air Resources Laboratory) was used to calculate the trajectories. Meteorological input data at 1 degree horizontal resolution was downloaded from the Global Data Assimilation System (GDAS) at https://www.ready.noaa.gov/archives.php. Air mass arrival sector was defined as the sector where the air mass had spent at least 80% of time during the 96-h back trajectory and three sectors were used: clean (280–30°), eastern (30–180°), and southern (180–280°) sector. Number of trajectories classified to these sectors in our analysis in July–August between 2012 and 2018 were 632, 605, and 1262, respectively. Rest of the trajectories did not spend the required 80% of time in any of the three sectors.

The effect of air mass arrival direction to the observed trend between OA mass loading and temperature was analyzed in a manner resembling concentration fields^44^: Area surrounding Hyytiälä was divided in 1° by 1° coordinate grid. The concentration and temperature observed at Hyytiälä at the arrival time of each trajectory were assigned to each 1° by 1° coordinate grid cell which the trajectory had passed through. Correlation coefficient between concentration and temperature values assigned to each grid cell was calculated. Only grid cells with at least 10 data points and *p*-value below 0.05 were included in Fig. 1c. This analysis showed that OA mass loading and temperature at Hyytiälä have a positive correlation despite of the prevailing direction of the air masses (Fig. 1c). Air mass source area (Supplementary Fig. 3a, b) for a summer (July–August) was constructed by calculating the number of times the trajectories passed through each 1° by 1° coordinate grid cell during the 96 h before arriving in Hyytiälä. As the coordinates were calculated for hourly intervals for each back trajectory, the number of times when air masses crossed a coordinate grid cell can be interpreted as linearly proportional to the time that air masses arriving to Hyytiälä spend in that grid cell during summer. Average OA mass loading and average temperature associated with trajectories arriving from different locations (i.e., “OA mass loading field” and “temperature field”) were also calculated (Supplementary Fig. 3c, d). The concentration and temperature observed at Hyytiälä at the arrival time of each trajectory were assigned to each 1° by 1° coordinate grid cell which the trajectory had passed through and the mean over the values assigned to each grid cell was calculated.

The time over land was calculated based on each hourly coordinate of the air mass trajectory. Cumulated rain along trajectory was calculated as a sum over the trajectory based on the rain data from HYSPLIT output.

### Remote sensing data

AERONET uses Cimel sun photometers which measure aerosol optical thickness (*τ*_a_) at 340, 380, 440, 500, 675, 870, and 1020 nm. Measurements are provided every 15 min during daytime. The spectral *τ*_a_ from AERONET is accurate to within ±0.01^45,46^. More detailed information on AERONET measurements is provided by Holben et al.^30^. In the analysis presented in this paper, cloud screened and quality assured level 2.0 direct sun and Spectral Deconvolution Algorithm^47^ (SDA) products were used from July to August 2012–2018. The daily medians of *τ*_a_ and Ångström exponent were calculated from the all points data product by requiring that within a day there were at least six observations (at least two between 4 and 7 UTC, at least two between 8 and 11 UTC, and at least two between 12 and 15 UTC). This was done to ensure that the daily median was representative. Only AERONET observations within 30 min of in situ observations were included to enable the best possible comparison between the different data sets. Furthermore, we screened out observations which had Ångström exponents (440–870 nm) below 0.75 as they were likely contaminated by clouds^48^.

The Moderate Resolution Imaging Spectroradiometer (MODIS)^31^ on board NASA’s Terra and Aqua satellites have been collecting information on Earth’s surface and atmosphere since 1999 and 2002, respectively. As the satellites are polar-orbiting and MODIS has a wide swath, they scan the entire surface of the Earth every 1–2 days. We used the level-3 MODIS gridded atmosphere daily global joint product (MYD08_D3, Collection 6.1) which contains average values of atmospheric parameters in 1° × 1° resolution. The parameters used in this analysis were aerosol optical thickness (*τ*_a_), cloud optical thickness (*τ*_c_), cloud effective radius (*r*_eff_), cloud water path, and cloud top temperature. The data were downloaded from NASA Giovanni (https://giovanni.gsfc.nasa.gov/giovanni/). For the *τ*_a_ analysis we selected the pixels over Hyytiälä (lat 61.85°, lon 24.29°) in July–August 2012–2018, whereas the analysis of the cloud properties was done over southern Finland (land regions between latitudes 60°–66° and longitudes 22°–30°). The size of the region was a compromise between sufficient amount of observations for statistical evaluation and representativity of the in situ observations done at Hyytiälä. As the selected region is covered with the same vegetation types, the observations done at Hyytiälä are a good proxy for the biogenic emissions in the whole study area. In practice, the collocation with in situ data was done by combining the daytime (7:00–14:00 UTC, centered on the Aqua overpass, minimum of 4 observations) medians of in situ observations with all the available satellite data pixels within the studied domain. To do the analysis with the most reliable cloud observations we limited the analysis to observations with *τ*_c_ between 5 and 50 and *r*_eff_ larger than 5 µm^49^. Only pixels which had more than 100 days of data were considered in the analysis.

### Calculation of radiative feedbacks

We estimated the regional direct radiative feedback (DRF) of the temperature-dependent *τ*_a_ component using the change of *τ*_a_ per temperature degree in the following equation (e.g., ^32^):

1 $${{{{{\rm{DRF}}}}}}={S}_{{{{{{\rm{rad}}}}}}}\varphi {\tau }_{a}\left(1-{C}_{c}\right){T}_{{{{{{\rm{atm}}}}}}}^{2}{\left(1-{R}_{{{{{{\rm{s}}}}}}}\right)}^{2}\left(2{R}_{{{{{{\rm{s}}}}}}}\frac{1-\omega }{{\left(1-{R}_{s}\right)}^{2}}-\beta \omega \right)$$

where *S*_rad_ is the incident solar radiation (398 W m^−2^) at the top of the atmosphere integrated over the 24-h day, *φ* is the mean daytime value of the secant of the solar zenith angle (1.82), *C*_c_ is the fractional cloud amount (0.0 for clear sky and 0.71 for all-sky), *T*_atm_ is the aerosol-free atmospheric transmission (0.76), *R*_s_ is the shortwave surface reflectance (0.12^50^), *ω* is the single scattering albedo (0.92^51^), and *β* is the up-scatter fraction (0.29^51^). The all-sky *C*_c_ for Hyytiälä was calculated from the cloud fraction data in July–August 2004–2018 available from MODIS-Aqua level-3 data. The region and season averaged *S*_rad_ and *φ* were calculated with the help of the tools in the LibRadtran package^52^. The DRF calculated from sun photometer and MODIS data are shown in Supplementary Table 2.

We estimated the cloud albedo feedback from the cloud optical thickness observations utilizing a division of the data according to OA mass loading and cloud water path. First, cloud albedo, *A*_c_ was estimated from the *τ*_c_ observations with a two-stream approximation following Bohren^53^:

2 $${A}_{c}=\frac{\frac{\left(1-g\right)}{{\cos }\theta }{\tau }_{{{{{{\rm{c}}}}}}}}{2+\frac{\left(1-g\right)}{{\cos }\theta }{\tau }_{{{{{{\rm{c}}}}}}}}$$

where *g* is the asymmetry factor (0.85^53^), and *θ* is the effective solar zenith angle (56.7°) calculated from the secant of the solar zenith angle (acos(*φ*)). The cloud albedo effect was calculated separately for each cloud water path category following Charlson et al.^54^:

3 $${{{{{\rm{CAE}}}}}}=-\frac{{{dA}}_{{{{{{\rm{c}}}}}}}}{d{\tau }_{{{{{{\rm{c}}}}}}}}{C}_{{{{{{\rm{c}}}}}}}{S}_{{{{{{\rm{rad}}}}}}}{T}_{{{{{{\rm{ft}}}}}}}^{2}\triangle {\tau }_{{{{{{\rm{c}}}}}}}=-\left(\frac{{A}_{{{{{{\rm{c}}}}}}}\left(1-{A}_{{{{{{\rm{c}}}}}}}\right)}{{\tau }_{{{{{{\rm{c}}}}}}}}\right){C}_{{{{{{\rm{c}}}}}}}{S}_{{{{{{\rm{rad}}}}}}}{T}_{{{{{{\rm{ft}}}}}}}^{2}({\tau }_{{{{{{\rm{c}}}}}},{{{{{\rm{h}}}}}}}-{\tau }_{{{{{{\rm{c}}}}}},{{{{{\rm{l}}}}}}})$$

where the *τ*_c,h_ and *τ*_c,l_ were the medians of the cloud optical thickness for high (>66th percentile) and low (<33rd percentile) aerosol loadings, respectively, in the corresponding cloud water path category and the *A*_c_ and *τ*_c_ were the medians over all values of cloud albedo and cloud optical thickness in the corresponding cloud water path category. The *T*^*2*^_ft_ is the free troposphere transmission without aerosols and its magnitude was estimated as 0.8 in Charlson et al.^54^. The final CAE estimate, −1.87 W m^−2^, was then calculated as an average of the individual CAE estimates weighted by the number of observations and statistical significance (1 for statistically significant difference, 0 otherwise) within each cloud water path category to ensure the representability of the average. The 95% confidence interval for CAE, −3.63 to −0.26 W m^−2^, was calculated by using the 2.5th and 97.5th percentiles of the *τ*_c,l_ and *τ*_c,h_ distributions in each category. In order to map the uncertainty range as completely as possible, the minimum (*τ*_c,h,2.5th_–*τ*_c,l,97.5th_) and maximum (*τ*_c,h,97.5th_–*τ*_c,l,2.5th_) differences between the *τ*_c,l_ and *τ*_c,h_ percentiles were used. This CAE corresponds to an average increase of 3.3 μg m^−3^ in OA mass loading, which was calculated as the average over the differences between the medians of the low and high OA loadings within each cloud water path category. Since the OA mass loading was shown to increase by 0.52 μg m^−3^ °C^−1^, the difference between the low and high OA categories (3.3 μg m^−3^) corresponds to a temperature difference of 6.3 °C. This gives a temperature-dependent cloud albedo feedback of −0.30 W m^−2^ °C^−1^ (95% confidence interval −0.58 to −0.04 W m^−2^ °C^−1^). Similar investigation was carried out by dividing the observations based on *N*_100_ (Supplementary Fig. 6) instead on OA mass loading. Similar investigation based on measured CCN concentration gave consistent results to *N*_100_, however, the differences were not statistically significant due to low number of data (see Supplementary Information).

### Radiative forcing of anthropogenic aerosols and comparison with BSOA-driven feedback

The shortwave effective radiative forcing (ERF) of present-day anthropogenic aerosols over the boreal region was calculated based on 14 contemporary climate models which have been contributed to the Radiative Forcing Model Intercomparison Project for CMIP6^55,56^. ERF is defined as the shortwave radiation flux difference at top of the atmosphere between simulated scenarios with present-day and preindustrial aerosols with climatological sea surface temperatures (SST) and sea ice distributions^57^. ERF over the boreal region in July–August was −3.50 W m^−2^ (mean over the 14 models, model values range from −5.36 to +1.04 W m^−2^).

To compare the BSOA-driven climate feedback to ERF, sum was taken of the cloud albedo feedback (−0.30 W m^−2^ °C^−1^) and the direct radiative feedback (−0.33 W m^−2^ °C^−1^) to obtain the combined feedback of −0.63 W m^−2^ °C^−1^ as different aerosol forcings can be considered to be additive^58^. For the direct radiative feedback, the sun photometer-based (AOD at 500 nm) estimate was used and it was assumed that the contribution comes from the cloud-free part of the sky only (by the term (1 − *C*_c_) in Eq. (1) for the all-sky condition estimate). The estimate for cloud albedo feedback takes into account that this feedback is induced over the cloud part of the sky (by the variable *C*_c_ in Eq. (3)). The significance of the feedback was estimated by dividing the combined BSOA-driven feedback per 1 °C on temperature increase (−0.63 W m^−2^) by the ERF over boreal area (−3.50 W m^−2^) which indicated that the BSOA-driven feedback per 1 °C of temperature increase would equal to 18% of the ERF of present-day anthropogenic aerosols over boreal region during summertime.

### Bayesian linear regression

The change in observed OA mass loading, *N*_100_ and *τ*_a_ with temperature (Figs. 1a, b and 2a) were calculated with Bayesian linear regressions. To avoid the possible underestimation of the slope, or regression to the mean, in the linear model parameters^59,60^, the Bayesian linear regression was carried out using a measurement error model that takes into account the uncertainties of both the dependent and independent variables. Before fitting the Bayesian measurement error model to the data both the dependent and independent variables were standardized. The uncertainties of the standardized dependent and independent variables were modeled as unknown parameters. The standard deviations of the uncertainties in all standardized variables were modeled with a standard normal distribution. Standard normal distributions were used as the prior distributions for the linear model parameters. A Markov chain Monte Carlo (MCMC) sampling scheme was used to draw 40,000 samples for the unknown parameters and conditional mean estimates for the linear model parameters were computed. The MCMC sampling was carried out using the STAN probabilistic programming language^61^.

### Multivariate mixed-effects model

The change in AO mass loading due to temperature-dependent processes was estimated with multivariate mixed-effects model^62^. The main idea of a mixed model is to estimate the variance–covariance structure of the data, and not only the mean of the response variable. Due to this, the model is not limited by the standard homogeneity and independency assumptions, required by general linear models, which are rarely fulfilled by atmospheric measurement data^27^. The model is also able to take account hierarchical structure of the data, formed by the sectors of air mass origin based on trajectory data. The explaining variables for OA mass loading in the model were: air mass arrival sector, time over land and cumulated rain from the back-trajectory data, in situ measured temperature and concentrations of NO_x_, BC, and CO, and the hour of the day. For this purpose, the eastern arrival sector was split in two (30–60° and 60–180°) to separate the air masses arriving above the Kola peninsula from the rest of the eastern sector and to improve the model performance. Correlation between the observed OA mass loading and the model prediction was 0.88. The significances of the explaining variables were tested by removing one explaining variable at the time from the model and comparing the Bayesian Information Criteria (BIC) value to the BIC value of full model (Supplementary Table 1, Supplementary Discussion).

## Supplementary information

Supplementary Information

## Data Availability

Field data on particle size distributions, meteorological variables, trace gases, and black carbon are available at https://smear.avaa.csc.fi/download (Variables: dN/dlogDp X nm (size distribution with each size class (X nm) provided separately, Air temperature 16.8 m, Wind direction, Global radiation 18/35 m, CO concentration 16.8 m, NO_x_ concentration X m (each measurement height X between 8.4 and 125 m separately), and BC 880 nm. ACSM data on aerosol composition are available at EBAS data base at http://ebas.nilu.no/ (Instrument type: aerosol_mass_spectrometer). AERONET and MODIS data are available at https://aeronet.gsfc.nasa.gov/ (Aerosol Optical Depth (AOD) with Precipitable Water and Angstrom Parameter, Spectral Deconvolution Algorithm (SDA) Retrievals—Fine Mode AOD, Coarse Mode AOD, and Fine Mode Fraction) and https://giovanni.gsfc.nasa.gov/giovanni/ (product DOI 10.5067/MODIS/MYD08_D3.061), respectively. HYSPLIT model is available at https://www.ready.noaa.gov/HYSPLIT_linux.php and the Global Data Assimilation System (GDAS) one-degree (GDAS1) archive data, used as meteorological input data for HYSPLIT in this study, is available for download as weekly datafiles from NOAA ARL ftp-server at https://www.ready.noaa.gov/archives.php (GDAS one-degree archive). The averaged and allocated data generated in this study and used for Figs. 1–3 are provided in the Source data file. Source data are provided with this paper.

## Source data

Source Data

## Code availability

Codes used in this study can be obtained from the corresponding author upon request.
