# Supplementary material for: Significance of the organic aerosol driven climate feedback in the boreal area
Source: Nat Commun. 2021 Sep 24;12:5637. doi: 10.1038/s41467-021-25850-7 (PMC8463617; doi:10.1038/s41467-021-25850-7)
Supplement: Supplementary file 1 — Supplementary Information [file 41467_2021_25850_MOESM1_ESM.pdf]

Supplementary Information:

Significance of the organic aerosol driven climate feedback in the boreal area

Taina Yli-Juuti, Tero Mielonen, Liine Heikkinen, Antti Arola, Mikael Ehn, Sini Isokääntä, Helmi-Marja Keskinen, Markku Kulmala, Anton Laakso, Antti Lipponen, Krista Luoma, Santtu Mikkonen, Tuomo Nieminen, Pauli Paasonen, Tuukka Petäjä, Sami Romakkaniemi, Juha Tonttila, Harri Kokkola and Annele Virtanen

A. Supplementary Discussion

A.1 Multivariate mixed effects model

To estimate the underlying effect of temperature on organic aerosol (OA) mass loading, we applied multivariate mixed effects model utilizing both back-trajectory and in situ observations (see Methods). The explaining variables in the model were air mass arrival sector, time over land and cumulated rain from the back-trajectory data, in situ observed temperature and concentrations of NO_x, black carbon (BC) and CO, and the hour of day. As air mass arrival sector was used as an explaining variable, only data for air masses that cloud be classified as arriving from one of the sectors (i.e. at least 80 % of the trajectory was within the limits of one of the sectors) could be included in this analysis. Correlation coefficient between observed OA mass loading and OA predicted with the model was 0.88. Based on the model, temperature was the most important variable to explain variation in OA mass loading. This was concluded by comparing the Bayesian Information Criteria (BIC) value between the full model and models where one explaining variable at a time was removed (Supplementary Table 1). The difference in BIC value between a model where temperature was removed and the full model was 11.6 %. Corresponding difference in BIC when removing BC from the model was 6.3 % making BC the second most important explaining variable. This possibly indicates an important, but not dominating, effect of biomass burning or anthropogenic contribution on observed OA mass loading. After temperature and BC, the next in the order of significance as an explaining variable were the hour of day, likely describing the effect of boundary layer dynamics, and the arrival sector of the air mass. The time that

the air mass had spent over land and the amount of rain the air mass had experienced during the 96-hour back trajectory had relatively low importance as explaining variables in the model (Supplementary Table 1).

The model was calculated also by using only data of air masses from one arrival sector at a time (i.e. three independent models, one per sector). Also in this case, for each of the three sectors, difference in BIC when removing temperature from the model was larger (16.5 % for clean, 8.2 % for eastern, 8.0 % for southern) compared to the difference in BIC when BC was removed (8.6 % for clean, 5.9 % for eastern, 7.1 % for southern). The leading role of temperature as an explaining variable for OA mass loading was especially clear for air masses arriving from the clean sector, while for air masses arriving from eastern and southern sectors difference in the importance between temperature and BC was smaller. The temperature dependent increase in OA mass loading estimated with these separate models for each sector were 0.23 $\mu g\ m^{-3}\ °C^{-1}$ for clean, 0.27 $\mu g\ m^{-3}\ °C^{-1}$ for eastern and 0.21 $\mu g\ m^{-3}\ °C^{-1}$ for southern sector. These values are close to the value 0.24 $\mu g\ m^{-3}\ °C^{-1}$ obtained with the model including data for all air mass arrival sectors. It should be noted that for the purpose of the statistical model, the eastern arrival sector was split in to two (30-60° and 60-180°) to separate the air masses arriving from northeast above the Kuala peninsula from the rest of the eastern sector and to improve the model performance. The results described above for the model on eastern sector refer to the sector 60-180°. The model results for air masses from the northeast (30-60°) were inconclusive due to low number of data.

These results from the multivariate mixed effects model support the conclusion that the anthropogenic pollution or biomass burning emissions are overall in a minor role in the observed increase of OA mass loading with temperature, and the trend is dominated by the increased biogenic secondary organic aerosol (BSOA) formation.

A.2 Solar radiation and cloudiness

Field data on OA mass loading were compared to aerosol optical thickness which was obtained from remote sensing observation only in clear sky conditions. To address the possible systematic influence of this, we divided the hourly data based on brightness parameter (BP) value (i.e. the ratio between the measured global radiation and the theoretical maximum global radiation intensity) in to "cloudy" (BP < 0.3) and "clear sky" (BP > 0.7) cases. Supplementary Fig. 8 shows that the on average temperature was lower in cloudy cases compared to clear sky cases and also the average concentrations differed between the two cases. However the increasing trend of OA mass loading with temperature is visible also for both brightness class separately.

The division of data in cloudy and clear sky cases demonstrates also the importance of changes in temperature dependent biogenic volatile organic compounds (BVOC) emissions in BSOA formation. Enhanced BSOA formation can be driven both by increased BVOC emissions and by increased oxidation due to the intensified UVB radiation. While UVB radiation intensity and temperature are positively correlated at the measurement site in summer, our analysis on cloudy and clear sky data (Supplementary Fig. 8) suggests that the increase in OA mass loading is dominated by the increased temperature dependent BVOC emissions over the increased oxidation. At a same temperature, both OA mass loading and number concentration of particles larger than 100 nm (N_{100}) tend to be lower at clear sky conditions compared to cloudy conditions. This is opposite to what would be expected if oxidation was driving the enhanced BSOA formation.

A.3 Cloud condensation nuclei concentration

Number concentration of particles larger than 100 nm calculated from the size distribution data were used as an approximation for cloud condensation nuclei (CCN) number concentration in this analysis. This approximation was necessary due to the lesser availability of CCN concentration data due to the

time resolution of four hours and gaps in the measurements. The CCN concentration measured with a CCN counter at 0.2 % supersaturation shows a temperature dependence similarly to N_{100} (Supplementary Fig. 7) although CCN concentration has a less steep slope with temperature compared to N_{100}. The correlation coefficient between the measured CCN concentration and N_{100} was 0.80.

A.4 Cloud properties and particle concentration

In the main text, cloud albedo feedback was calculated based on cloud properties and OA mass loading. The cloud albedo feedback was calculated in similar manner also based on number concentration of particles larger than 100 nm (N_{100}) instead on OA mass loading (Supplementary Fig. 6). In this case the data in each cloud water path category was divided to low (< 340 cm^{-3}) and high (> 633 cm^{-3}) N_{100} classes. The differences between the N_{100} classes were statistically significant (t-test with 95 % confidence level) in the six highest cloud water path categories. From the statistically significant changes in cloud optical thickness (τ_c) we estimated the corresponding change in cloud albedo using Eq. (2). Then, using this calculated change in albedo in Eq. (3) we estimated the CAE which was -2.59 W m^{-2} (95 % confidence interval -4.51 - -0.19 W m^{-2}). The albedo change corresponds to a median increase of 568 cm^{-3} in the N_{100}. The change in N_{100} was calculated as the average difference between the medians of the low (233 cm^{-3}) and high (808 cm^{-3}) N_{100} classes in the cloud water path categories. As the N_{100} was shown to increase by 88 cm^{-3} °C^{-1}, the difference between the low and high N_{100} (568 cm^{-3} °C^{-1}) corresponds to a temperature difference of 6.4 °C. Consequently, the temperature dependent cloud albedo feedback is -0.40 W m^{-2} °C^{-1} (95 % confidence interval -0.70 - -0.03 W m^{-2} °C^{-1}) which is in the same range but slightly stronger than the estimate based on OA mass loadings.

Similar investigation was attempted also using measured CCN number concentration instead of N_{100}. The results based on CCN number concentration were consistent with those derived using N_{100}, however the differences were not statistically significant due to low number of data.

Supplementary Table 1. Significances of the explaining variables in variability of OA mass loading. Bayesian information criterion (BIC) value and the relative difference in BIC (diff. %) compared full model when one variable at a time is removed from the model. CO, NO_x and black carbon (BC) concentration and temperature are in-situ observations. Cumulated rain, time over land and arrival sector were calculated from the trajectory data. Results are presented for model applied for the whole data and for model applied separately for data from the different air mass arrival sectors.

Removed variable	All directions		Clean sector		Eastern sector		Southern sector	
	BIC	diff. %	BIC	diff. %	BIC	diff. %	BIC	diff. %
none	7965.8	N/A	2080.3	N/A	1093.3	N/A	4622.0	N/A
Temperature	9008.5	11.6	2492.0	16.5	1191.1	8.2	5024.1	8.0
BC	8505.5	6.3	2275.4	8.6	1161.2	5.9	4973.9	7.1
Hour of day	8432.8	5.5	N/A	N/A	N/A	N/A	N/A	N/A
Arrival sector	8274.5	3.7	N/A	N/A	N/A	N/A	N/A	N/A
CO	8059.4	1.2	2073.0	-0.4	1158.5	5.6	4637.5	0.3
Time over land	8004.0	0.5	2124.5	2.1	1081.5	-1.1	4616.2	-0.1
Cumulative rain	8002.8	0.5	2078.8	-0.1	1083.0	-0.9	4637.6	0.3
NO_x	7989.8	0.3	2127.6	2.2	1099.9	0.6	4613.0	-0.2

Supplementary Table 2. Direct radiative feedback and cloud albedo feedback. Direct radiative feedback (DRF) was calculated from sun photometer and MODIS data for both clear and all sky conditions. Cloud albedo feedback was calculated from the MODIS data by grouping the data either based on the OA mass loadings or based on N_{100}.

	Feedback	95 % credible interval / 95 % confidence interval of the feedback
DRF from sun photometer (at 500 nm), clear sky	-1.15 W m^{-2} $°C^{-1}$	-2.49 - -0.47 W m^{-2} $°C^{-1}$
DRF from sun photometer at 500 nm, all sky	-0.33 W m^{-2} $°C^{-1}$	-0.72 - -0.14 W m^{-2} $°C^{-1}$
DRF from MODIS, clear sky	-1.56 W m^{-2} $°C^{-1}$	-4.57 - -0.22 W m^{-2} $°C^{-1}$
DRF from MODIS, all sky	-0.45 W m^{-2} $°C^{-1}$	-1.33 - -0.06 W m^{-2} $°C^{-1}$
Cloud albedo feedback, OA	-0.30 W m^{-2} $°C^{-1}$	-0.58 - -0.04 W m^{-2} $°C^{-1}$
Cloud albedo feedback, N_{100}	-0.40 W m^{-2} $°C^{-1}$	-0.70 - -0.03 W m^{-2} $°C^{-1}$

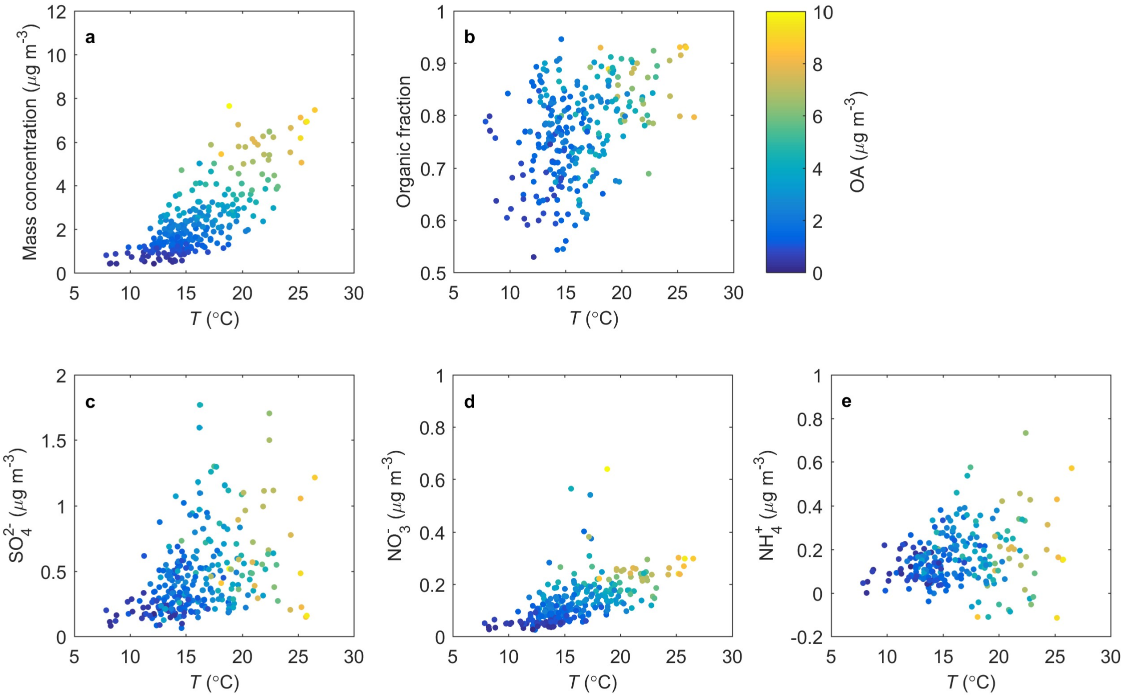

Supplementary Figure 1. Particle mass concentration and composition as a function of temperature (*T*). a) Total particle mass concentration calculated from size distribution data with assumed density of 1000 kg m^{-3} as a function of temperature. b) Organic mass fraction measured with ACSM. c) Sulfate (SO_4^{2-}), d) nitrate (NO_3^-) and e) ammonium (NH_4^+) mass concentration measured with ACSM. Daily medians from summer (July-August) with color indicating organic aerosol (OA) mass loading.

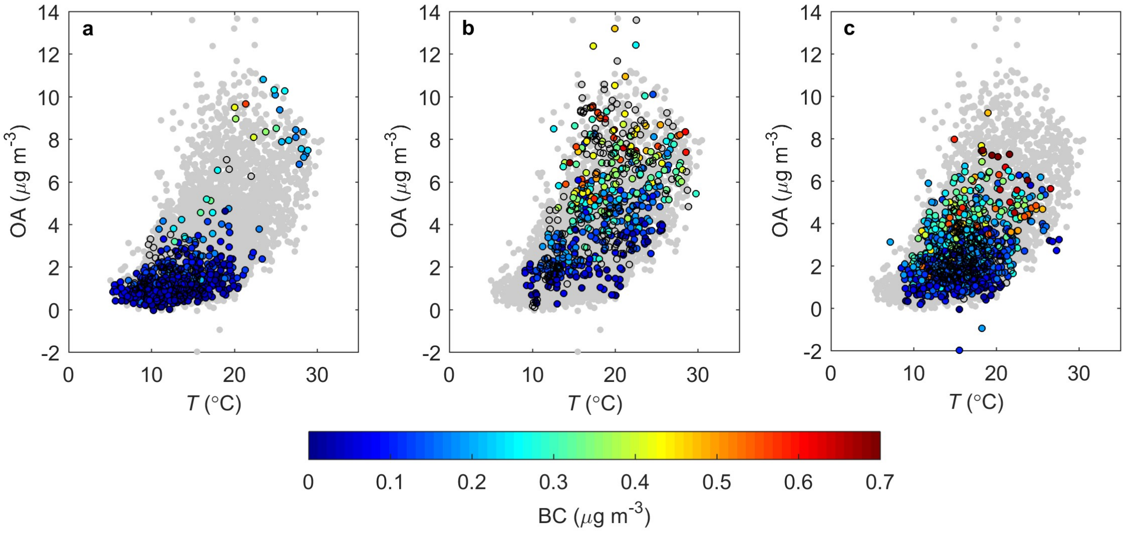

Supplementary Figure 2. Organic aerosol (OA) mass loading separated by origin of air mass. OA mass loading observed at Hyytiälä as a function of temperature (*T*) for air masses arriving from a) clean, b) eastern and c) southern sector. The circles colored with black carbon (BC) concentrations show the data associated with the trajectories that had spent at least 80% of the time in the corresponding sector. All hourly data from July-August are shown with grey circles in each subfigure for reference.

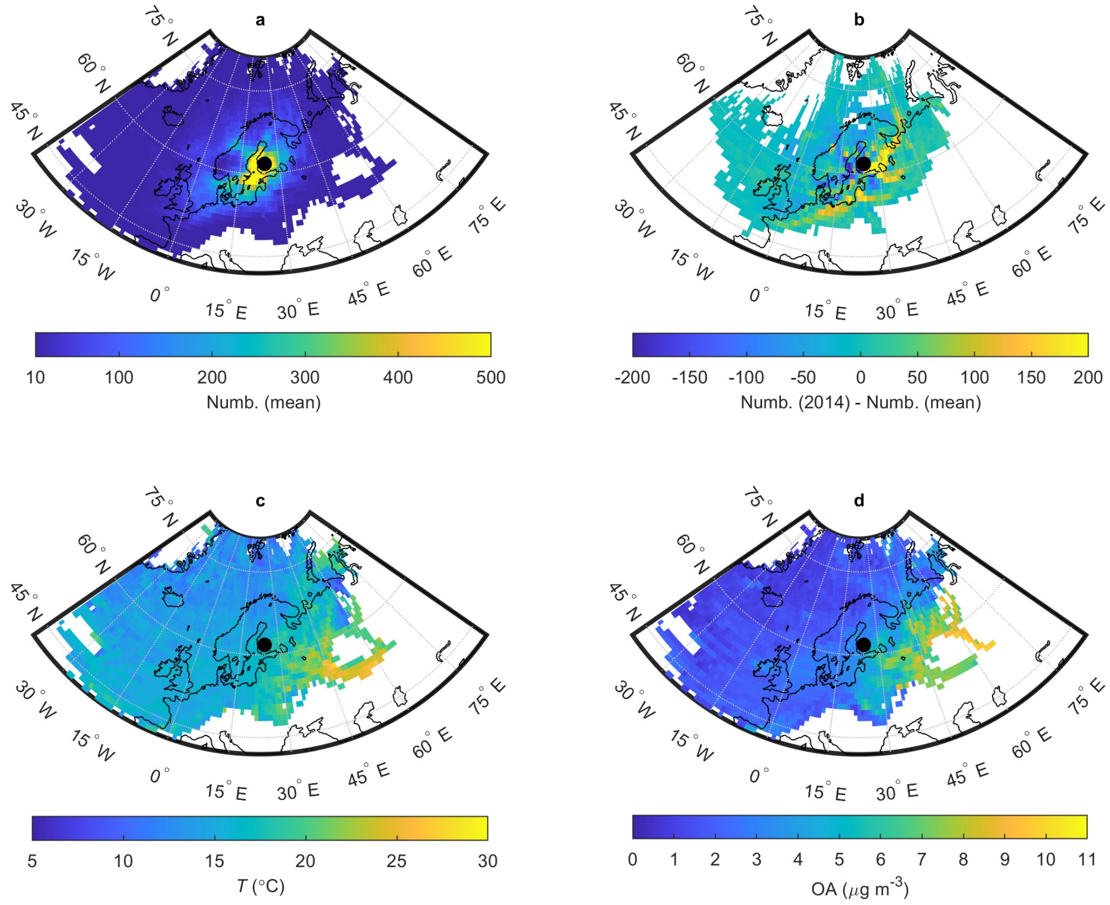

Supplementary Figure 3. Origin of air masses and associated temperature and organic aerosol (OA) mass loading. a) Mean of air mass source area for July-August over years 2012-2018, i.e. the average number of times trajectories passed through each 1° by 1° coordinate grid cell per summer (Numb. (mean)). b) The difference in source area between warm summer 2014 (Numb. (2014)) and the average over summers 2012-2018 (Numb. (mean)). c) Average temperature (*T*) and d) average OA mass loading associated with trajectories arriving from different locations over summers 2012-2018.

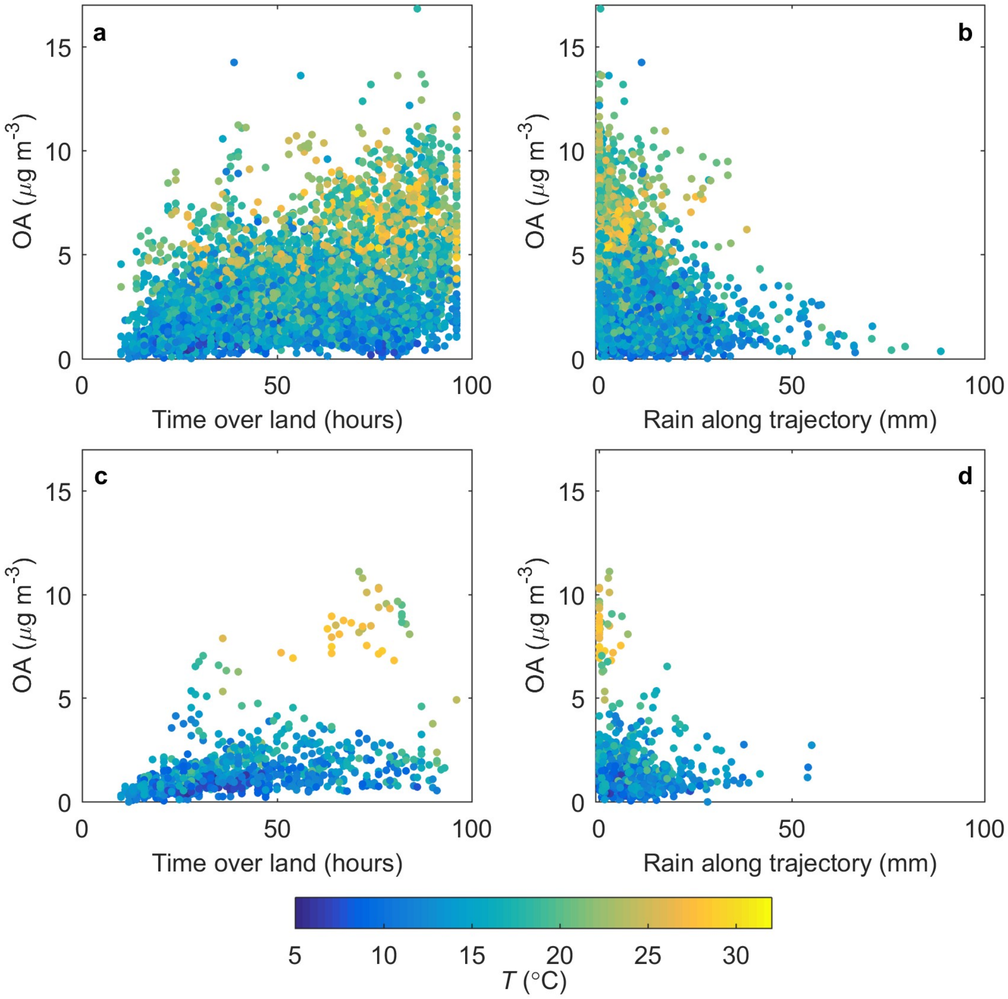

Supplementary Figure 4. Organic aerosol (OA) mass loading and the trajectory history. OA mass concentration observed at Hyytiälä at the time of arrival of a calculated back trajectory as a function the time the trajectory has spent over land (a, c) and the rain in the air mass (b, d) along the 96-hours back trajectory. Color indicates the temperature (*T*) observed at Hyytiälä. Data is shown for all trajectories (a, b) and separately for trajectories arriving from the clean sector (c, d).

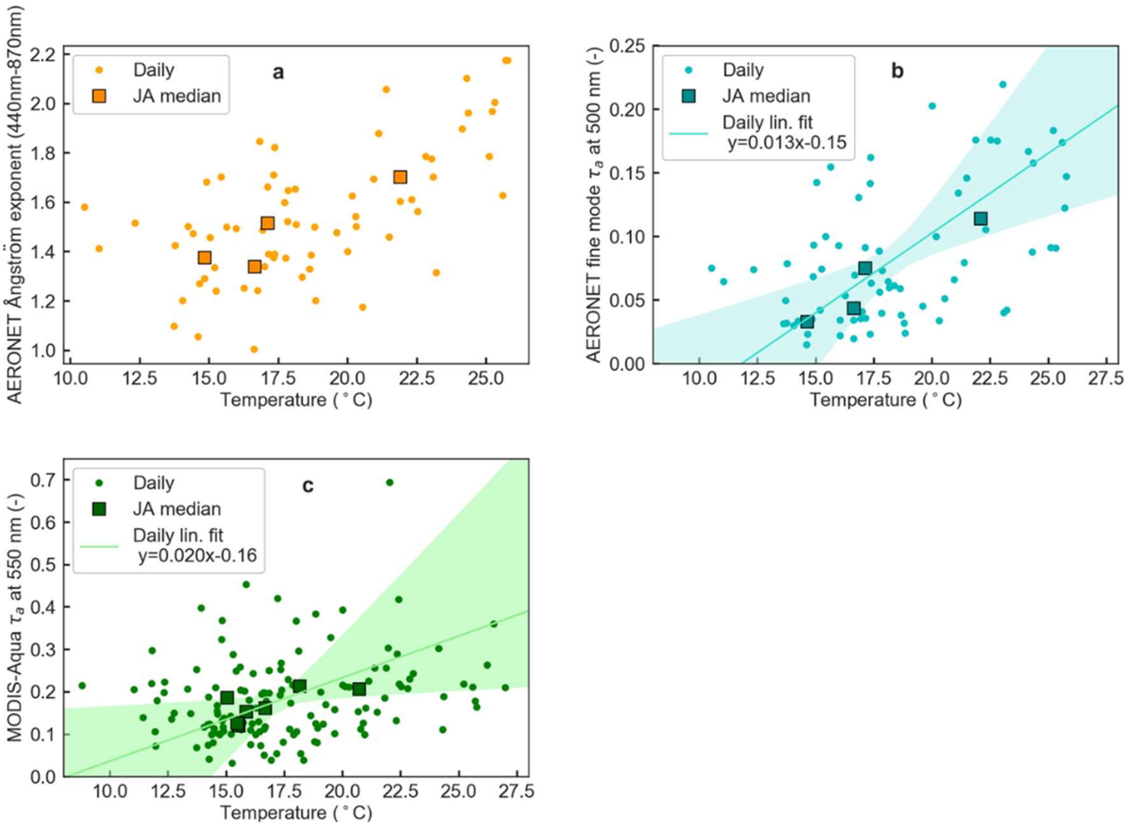

Supplementary Figure 5. Sun photometer and MODIS observations at Hyytiälä. a) Ångström exponent (440-870 nm) measured with a sun photometer in Hyytiälä as a function of temperature. b) Fine mode aerosol optical thickness (τ_a) at 500 nm measured with a sun photometer in Hyytiälä as a function of temperature. The slope of the linear fit is 0.013 °C^{-1} (95 % credible interval 0.005 - 0.025 °C^{-1}). c) Aerosol optical thickness at 550 nm measured with a MODIS-Aqua over Hyytiälä as a function of temperature. The slope of the linear fit is 0.020 °C^{-1} (95 % credible interval 0.003 - 0.058 °C^{-1}).

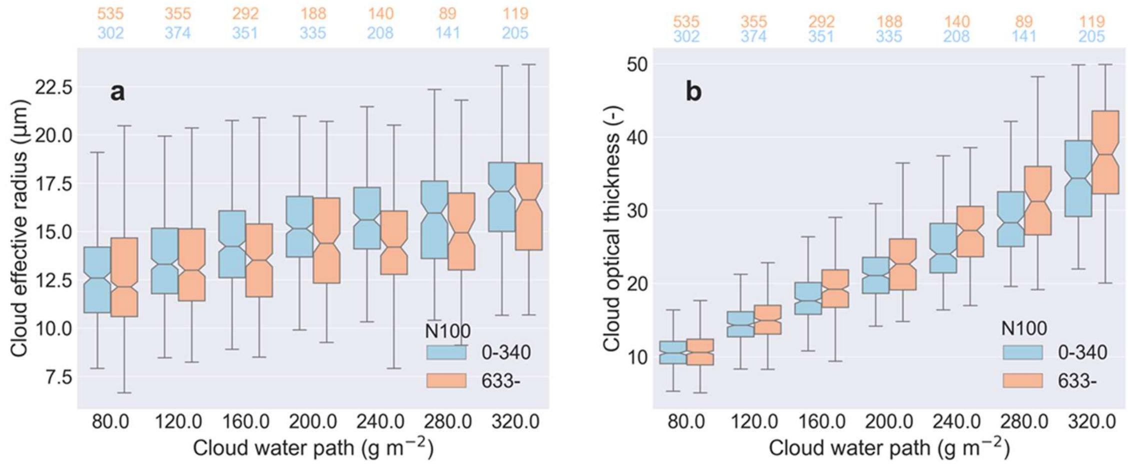

Supplementary Figure 6. Cloud properties and N_{100}. a) Cloud effective radius and b) cloud optical thickness divided based on level of cloud water path. Data is divided to low (< 33rd percentile (340 cm^{-3}), blue) and high (> 66th percentile (633 cm^{-3}), red) number concentrations of particles larger than 100 nm. The box shows the quartiles of the dataset while the whiskers show the rest of the distribution, except for points that are determined to be “outliers” using a method that is a function of the inter-quartile range. The notch in the box displays the confidence interval around the median. The blue and red numbers above each figure indicate the number of data points in each box.

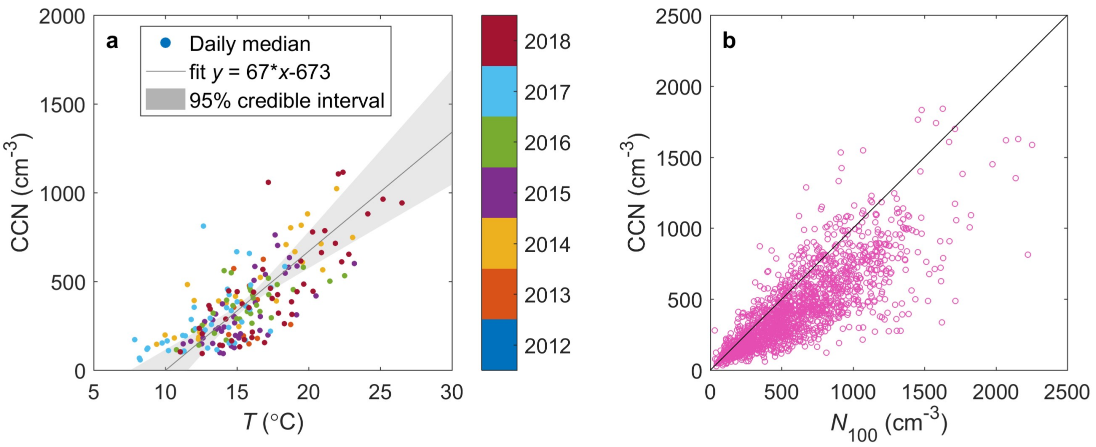

Supplementary Figure 7. Field observations on cloud condensation nuclei concentration (CCN). a) Measured CCN number concentration at 0.2 % supersaturation as a function of temperature (*T*). Daily medians from summer (July-August) are shown with color indicating the year. Color scale corresponds to Fig. 1 although CCN data for 2012 is missing. Fitted Bayesian linear regression model is shown with gray line and the shaded area indicates the 95% credible interval for the fitted linear model. b) Measured CCN concentration as a function of number concentration of particles larger than 100 nm (N_{100}). Hourly data is presented with pink circles and 1:1 line is shown with black.

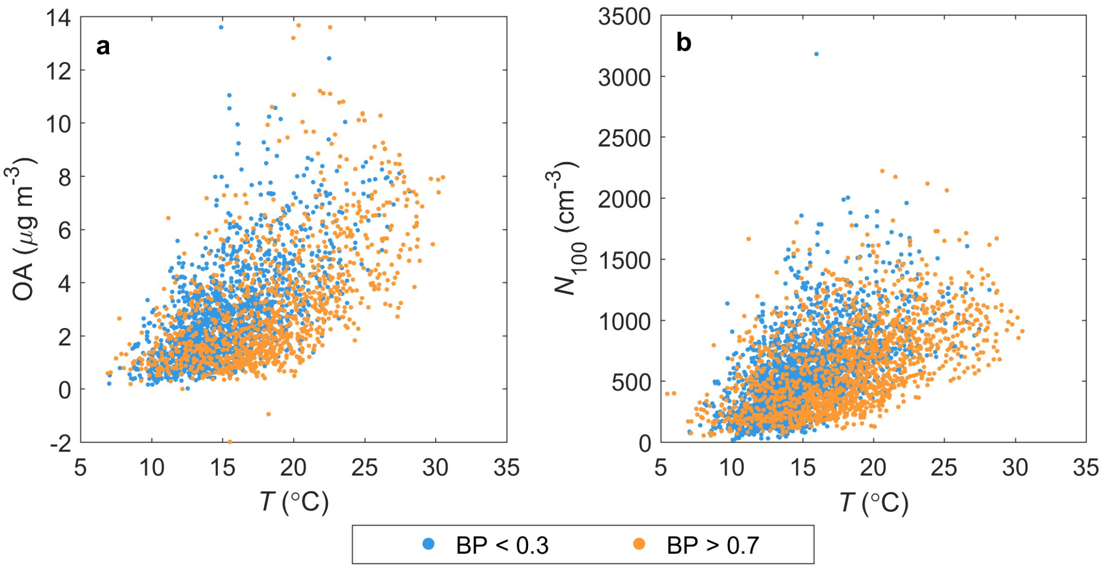

Supplementary Figure 8. Organic aerosol (OA) mass loading at cloudy and clear sky conditions. Hourly means of July-August a) OA mass loading and b) number concentration of particles larger than 100 nm (N_{100}) as a function of temperature (T) for "cloudy" (brightness parameters BP < 0.3, blue) and "clear sky" (BP > 0.7, yellow) cases. Night time (determined here as time when sun is less than 1° over the horizon) data were removed from the analysis. At a same temperature, both OA mass loading and N_{100} tend to be lower at clear sky conditions compared to cloudy conditions.
